# Computational mining of B cell receptor repertoires reveals antigen-specific and convergent responses to Ebola vaccination

**DOI:** 10.3389/fimmu.2024.1383753

**Published:** 2024-07-08

**Authors:** Eve Richardson, Sagida Bibi, Florence McLean, Lisa Schimanski, Pramila Rijal, Marie Ghraichy, Valentin von Niederhäusern, Johannes Trück, Elizabeth A. Clutterbuck, Daniel O’Connor, Kerstin Luhn, Alain Townsend, Bjoern Peters, Andrew J. Pollard, Charlotte M. Deane, Dominic F. Kelly

**Affiliations:** ^1^ Department of Statistics, University of Oxford, Oxford, United Kingdom; ^2^ Oxford Vaccine Group, Department of Pediatrics, University of Oxford, Oxford, United Kingdom; ^3^ La Jolla Institute for Immunology, La Jolla, CA, United States; ^4^ Weatherall Institute for Molecular Medicine, University of Oxford, Oxford, United Kingdom; ^5^ Divisions of Allergy and Immunology, University Children’s Hospital and Children’s Research Center, University of Zurich (UZH), Zurich, Switzerland; ^6^ Janssen Vaccines and Prevention, Leiden, Netherlands; ^7^ NIHR Oxford Biomedical Research Centre, Oxford University Hospitals NHS Foundation Trust, Oxford, United Kingdom

**Keywords:** vaccination, BCR - B cell receptor, BCR-Seq, Ebola (EBOV), monoclonal abs, prediction model

## Abstract

Outbreaks of Ebolaviruses, such as Sudanvirus (SUDV) in Uganda in 2022, demonstrate that species other than the Zaire ebolavirus (EBOV), which is currently the sole virus represented in current licensed vaccines, remain a major threat to global health. There is a pressing need to develop effective pan-species vaccines and novel monoclonal antibody-based therapeutics for Ebolavirus disease. In response to recent outbreaks, the two dose, heterologous Ad26.ZEBOV/MVA-BN-Filo vaccine regimen was developed and was tested in a large phase II clinical trial (EBL2001) as part of the EBOVAC2 consortium. Here, we perform bulk sequencing of the variable heavy chain (VH) of B cell receptors (BCR) in forty participants from the EBL2001 trial in order to characterize the BCR repertoire in response to vaccination with Ad26.ZEBOV/MVA-BN-Filo. We develop a comprehensive database, EBOV-AbDab, of publicly available Ebolavirus-specific antibody sequences. We then use our database to predict the antigen-specific component of the vaccinee repertoires. Our results show striking convergence in VH germline gene usage across participants following the MVA-BN-Filo dose, and provide further evidence of the role of IGHV3–15 and IGHV3–13 antibodies in the B cell response to Ebolavirus glycoprotein. Furthermore, we found that previously described Ebola-specific mAb sequences present in EBOV-AbDab were sufficient to describe at least one of the ten most expanded BCR clonotypes in more than two thirds of our cohort of vaccinees following the boost, providing proof of principle for the utility of computational mining of immune repertoires.

## Graphical Abstract

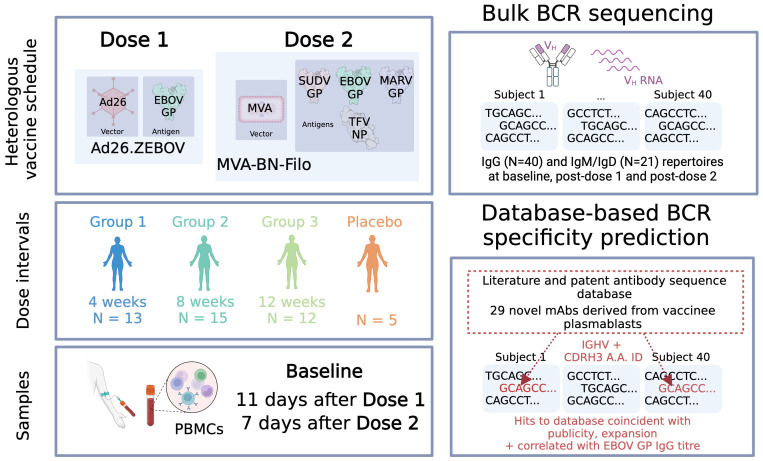

## Introduction

Ebolaviruses are highly infectious zoonotic filoviruses which can cause severe hemorrhagic fever in humans, referred to as Ebolavirus disease (EVD). EVD can have mortality rates of up to 90% (1). There are six species currently classified within the Ebolavirus genus: *Zaire ebolavirus* (EBOV), *Sudan ebolavirus* (SUDV), *Bundibugyo ebolavirus* (BDBV), *Tai Forest ebolavirus* (TFV), *Reston virus* (RESTV) and the most recently described *Bombali ebolavirus* (BOMV). All but Reston and Bombali virus have been associated with severe disease in humans (2, 3). Only three species (EBOV, SUDV, BDBV) have caused outbreaks, with EBOV and SUDV in particular responsible for tens of thousands of deaths in over thirty separate outbreaks in West and equatorial Africa since 1976 (4, 5). Outbreaks continue to occur with regularity, and there have been three distinct Ebolavirus outbreaks in the Democratic Republic of Congo (DRC) between May 2018 and November 2020, an outbreak in Guinea in 2021, and again in the DRC between April and June of 2022. The most recent outbreak was in Uganda from September 2022 to January of 2023.

The 2013–16 Ebola virus outbreak in the DRC was the largest to date, causing in excess of 28,000 cases and 11,000 deaths (6). This epidemic expedited human safety and efficacy testing of Ebola vaccine candidates (7, 8) and the first Ebola virus vaccine, ERVEBO, was approved for use in 2019. ERVEBO is a replication-competent vesicular stomatitis virus (rVSV) based vaccine, and is currently the only FDA-approved vaccine used to immunize at-risk individuals during active outbreaks. ERVEBO is monovalent, only containing the surface glycoprotein of *Zaire ebolavirus*, and efficacy has only been demonstrated for this species. In addition to ERVEBO, a heterologous two-dose vaccination regimen using an adenovirus viral vector expressing Zaire ebolavirus glycoprotein (Ad26.ZEBOV) and an Ankara vector based vaccine expressing the Zaire, Ebola and Sudan ebolavirus glycoproteins along with Tai Forest virus nucleoprotein (MVA-BN-Filo), showed safety and immunogenicity in clinical trials and was licensed for prophylactic use in the European Union in 2020 (9–14). Both vaccines are licensed as monovalent vaccines against *Zaire ebolavirus*.

B cells isolated from convalescent human participants and vaccinees (with both ERVEBO and ChAD3.EBOV/MVA-BN-Filo) have been an important source of therapeutic monoclonals for Ebolavirus. Two monoclonal antibody (mAb)-based immunotherapeutics, Inmazeb and Ebanga/mAb114, are currently FDA approved for the treatment of EVD, however, these only confer moderate protection (15–17). Ebanga/mAb114 was discovered in memory B cells of a survivor of the 1995 Kikwit EVD outbreak (18) and the two component mAbs of MBP134AF were discovered in a survivor of the 2014 EVD outbreak (19, 20). These mAbs are among hundreds discovered in EVD survivors (18, 19, 21–29). Most recently, Chen and colleagues conducted a large-scale sequencing study of a survivor of the 2014 EVD outbreak in Nigeria, estimating over 20,000 EBOV GP-specific clonal lineages within the memory B cell repertoire in just this single participant (30). Among antibody discovery efforts in vaccinees, Rjial and colleagues identified 82 anti-EBOV GP monoclonals from the memory B cells and plasmablasts of participants vaccinated with the ChAD3.EBOV/MVA-BN-Filo vaccine in 2019, while Ehrhardt and colleagues identified 94 anti-EBOV GP monoclonals from rVSV vaccinees (31, 32). As part of the Viral Hemorrhagic Fever Immunotherapeutics Consortium, Saphire and colleagues studied 171 mAbs (of which 102 were human-derived) in the context of the epitopes targeted, neutralization and protection in a mouse model (33). Survivor-derived mAbs and derivatives thereof currently constitute the majority of current immunotherapies for Ebolavirus. While several vaccinee-derived mAbs have demonstrated protection in mice and NHPs, none are currently in the clinic.

In addition to acting as a source of monoclonal antibodies, B cells and their receptor repertoires provide an important window into the response to Ebolavirus infection and vaccination. A recurrent theme in B cell receptor (BCR) repertoire studies in Ebolavirus and in infectious disease more generally, is the concept of public clonotypes, i.e., groups of related BCR sequences observed in multiple independent participants. Studies of the BCR repertoire in convalescence, and of EBOV-GP specific monoclonal antibodies have highlighted a number of public responses, including usage of IGHV3–13 in antibodies which target the GP1 region of the glycoprotein, IGHV3–15/IGLV1–40-encoded antibodies which target the receptor binding region (RBR), and IGHV1–69 and IGHV1–2 antibodies, which may be important in the early antiviral response (25, 30). Sequencing of four individuals vaccinated with ERVEBO identified a number of public clonotypes shared between the four vaccinees (32). Recently, Chen et al. curated a database of EBOV-specific antibodies from 12 either vaccinated or infected individuals across five studies (30). However, there are currently no publicly available databases where these sequences are compiled.

In the present work, we examined the B cell receptor repertoire response of participants in the Ad26.ZEBOV/MVA-BN-Filo trial. We generated bulk BCR repertoires from forty-five individuals enrolled in the trial, split into three groups according to timing of dose 2 administration, at baseline, after the monovalent dose 1 and the multivalent dose 2. We used our database to computationally annotate the likely antigen-specific component of these repertoires.

## Materials and methods

### Compilation of EBOV-AbDab

Publications describing Ebolavirus specific monoclonal antibodies were identified from the Immune Epitope database (a database of experimental B and T-cell epitope data by searching for B cell assay data with Ebolavirus as the Epitope Organism. EBOV-specific sequences from patents were retrieved from PLAbDab via searching for the word Ebolavirus (34, 35). Germline gene assignment and identification of CDR3s for the identified antibodies were calculated using IgBLAST and the appropriate IMGT database (human, mouse or macaque) (36–38). In the absence of available nucleotide sequence data, we curated amino acid sequence and used IgBLAST-aa to assign IGV genes and ANARCI to assign IGJ genes (36, 39). In the absence of nucleotide or amino acid sequences, germline genes and CDRH3 and CDRL3 sequences were collected as reported in the original publications. Binding data and neutralization data was collected where available for each antibody as well as, if available, binding to sGP. To create a non-Ebolavirus specific baseline for our antibody specificity predictions, we used two databases: Human CoV-AbDab, filtering for human antibodies based on the Heavy V Gene attribute (dated 13/6/23) and the IEDB (dated 13/6/23), after removing Ebolavirus-specific mAb sequences (34, 40). This resulted in 10,741 and 2,022 entries respectively. We also compared IGHV and IGKLV gene frequencies to a database of HIV antibodies, CATNAP (41). We filtered for IGHV and IGKLV genes and CDRH3s resulting in 394 entries.

### Isolation of mAbs from plasmablasts

Antibodies were isolated by FACS sorting, PCR and antibody variable gene cloning of a single B cell plasmablast from six vaccinated human individuals using the previously described methods (Rijal et al., 2019). Briefly, PBMC were incubated with a cocktail of antibodies to CD3 (PB; UCHT1; BD PharMingen), CD20 (APC-H7; 2H7; BD PharMingen), CD19 (FITC; H1B19; BD PharMingen), CD27 (PE-Cy7, M-T271; BD PharMingen), CD38 (PE-Cy5, HIT2; BD PharMingen) and IgG (BV605, G18–145; BD PharMingen). For some sorts, Ebola GP protein (10 μg/mL) and a known biotin-labeled anti-MLD antibody (10 μg/mL) were used to sort antigen specific B cell plasmablasts. Single cells with the phenotype of CD3^-^ CD20^-/low^, CD19^+^, CD27^++^, CD38^++^, IgG^+^ were sorted on a FACS Aria III cell sorter (BD Biosciences). Single cells were sorted into 96-well PCR plates containing lysis buffer followed by single cell RT-PCR. Nested PCR was slightly modified to existing methods. Overlapping bases (approx. 20 nucleotides) were added on to existing 5′ and 3′ primers without interfering the restriction sites, which could be used as a back-up, to enable digestion free Gibson cloning. PCR products were purified in a QIAGEN 96-well system and the inserts were assembled with restriction enzyme-digested plasmids in the Gibson mix (NEB). Two μL of assembled product was used to transform 10 μL DH5α E. Coli (NEB, C2987) in 96-well plates. Three colonies for each heavy and light chain were grown in a 96-well plate format and purified using QIAGEN Turbo 96 miniprep kit. Plasmids were eluted using 100 μL TE buffer.

### Expression and purification of antibody

Antibodies were expressed in ExpiCHO cells (Thermo Fisher) by co-transfection with heavy and light plasmids. Antibodies were purified from harvested cell supernatant using MabSelect SuRe (GE Healthcare, 17–5438-01). The column was washed with Tris buffered saline (TBS) and eluted with sodium citrate buffer pH 3.0 – 3.4. Elution pools were neutralized with 2 M Tris/HCl pH 8.0 and absorbance read at 280 nm. Samples were then buffer exchanged into PBS pH 7.4 using 10ml Zeba spin desalting columns, 7K MWCO (Thermo Fisher 89893).

### EBL2001 vaccine trial

EBL2001 was a heterologous two-dose randomized, double-blind, placebo-controlled, phase 2 trial of a new Ebolavirus vaccine, performed by the EBOVAC2 consortium (9, 10, 12). Dose 1 of the vaccination regimen is a replication-deficient adenovirus type 26 vector-based vaccine (Ad26.ZEBOV), encoding Zaire Ebola virus glycoprotein, and the dose 2 vaccination is a non-replicating, recombinant, modified Vaccinia ankara (MVA) vector-based vaccine, encoding glycoproteins from Zaire Ebola virus, Sudan virus, and Marburg virus, and nucleoprotein from the Tai Forest virus. Four hundred twenty three participants were enrolled and randomly assigned to the three different regimes (Groups 1, 2 and 3). Dose 1 administration consisted of either Ad26.ZEBOV or placebo, then this was followed by either MVA-BN-Filo or placebo as dose 2 at 28 (group 1), 56 (group 2), or 84 (group 3) days later.

### Samples for BCR sequencing

Peripheral blood was taken from 45 participants enrolled in the EBL2001 trial. Forty participants received the Ad26.ZEBOV dose 1 and MVA-BN-Filo dose 2, while five had received a placebo at both doses. Subjects were selected according to sample availability. Thirteen participants were from interval regimen group 1, 15 from interval regimen group 2, and 12 from interval regimen group 3. Samples were taken prior to vaccination, referred to as Baseline, 11 days following dose 1 referred to as Post-dose 1, and 7 days post-dose 2 referred to as Post-dose 2. 42 of these 45 participants (38/40 vaccinees; 4/5 control participants) were white, with the remainder of Asian (1), mixed (1) or Unknown ethnicity. Twenty-five participants were female and 20 were male. The average age was 42.5, 39, 37.4 and 36.6 years in Group 1, 2, 3 and the Placebo cohort each.

### BCR sequencing

PBMCs were isolated via Ficoll-Paque density centrifugation. RNA was extracted using Qiagen RNeasy kit. RT-PCR was performed separately with either IgG, IgA and IgE (all 45 participants) or IgM and IgD primers (21 participants), incorporating unique molecular identifiers (UMIs). V_H_ cDNA was amplified using a mix of IGHV region primers and Illumina adapter primers as per previous work (42). Samples were multiplexed via combinatorial dual indexing.

### Processing of BCR-seq data

BCR-seq data was processed using the Immcantation toolkit (v. 4.4.0) (43, 44). Samples were demultiplexed using the i5 and i7 Illumina indices. A quality filter was applied using *FilterSeq* with a quality cut-off of 30; paired-end reads were joined and merged, and consensuses built according to their UMIs. IgBlast was used to perform germline gene assignment using the *AssignGenes* wrapper with a standard IMGT human germline database, and isotype subtype annotated was performed using *stampy* (36, 45). Sequences were grouped into clonotypes within participant and time points, across time points within the same participant using the *DefineClones* module, with a junctional amino acid identity threshold of 90%. There are multiple clonotype definitions in use: we selected 90% as intermediate in the common range of 80 – 100%. To combat possible index hopping despite dual indexing, the presented analyses consider only UMIs supported by at least two reads or sequences supported by at least two reads. Where sequence or clone abundance is mentioned, this refers to the number of unique UMIs. Without the sequence count filter, we obtained on average 19,257.1 ± 2,832.9 and 99,781.5 ± 10,607.4 sequences per sample; applying this filter resulted in 5,404.2 ± 623.5 sequences per sample and 40,670.8 ± 3,616.7 unique sequences per sample respectively.

### Participant EBOV-GP IgG titers

Humoral immunogenicity assessments were carried out with serum from participants and Total IgG Ebola virus glycoprotein-specific binding antibody concentrations were measured by use of an Ebola virus glycoprotein Filovirus Animal Non-Clinical Group ELISA at Q^2^ Solutions Laboratories (San Juan Capistrano, CA, USA). Data and methods previously published in Pollard et al. (2021) (9). IgG titers for EBOV GP were measured at baseline and 21 days post-dose 2 for 42/45 participants (37 vaccinee and 5 control).

### Competition ELISA

mAb114 and mAb040 which bind non-overlapping epitopes on the Ebola glycoprotein, (the receptor binding region and the glycan cap respectively), were biotinylated using the EZ-Link™ Sulfo-NHS-Biotinylation Kit Biotin-labeled mAb was mixed with unconjugated blocker mAb in a 50-fold excess and they were let to compete for binding to the EBOV-GP on the cell surface. The binding by the biotin-labelled mAb was detected using streptavidin-HRP and TMB peroxidase substrate (Seracare, Cat No. 5120–0076). The reaction was stopped with 1M H_2_SO_4_ and the absorbance at 450 nm was read using the CLARIOstar plate reader. The data is shown as a percentage of biotinylated mAb binding compared to maximal binding (non-overlapping mAb blocker).

### Calculation of immune repertoire parameters

Custom Python scripts were used to calculate parameters such as Gini index, median IGHV identity (% nucleotide identity to the assigned IGHV allele) and to identify expanded and convergent clonotypes. The formula for Gini index is as per *Formula 1*. For IGHV fold changes, fold changes are calculated with a pseudocount of 1.


$$G=\sum_{i=1}^{n}(2i-n-1)x_{i}/n\sum_{i=1}^{n}x_{i}$$

Formula 1: Gini index

### Statistical methods

Non-parametric methods are used, i.e. for paired tests, Wilcoxon rank-sum test (implemented with *scipy.stats.wilcoxon*), and for non-paired tests, Mann-Whitney U-test (implemented with *scipy.stats.mannwhitneyu*) (46). Multiple testing correction is performed via the Benjamini-Hochberg method within *statsmodels.multitests.multipletests* (47). For the IGHV gene comparison to reduce the number of tests performed, repeated measures ANOVA is used prior to *post-hoc* Mann-Whitney U-testing (using *statsmodels.stats.anova.AnovaRM*). For the correlation analysis, Spearman’s rank correlation coefficient was calculated with *scipy.stats.spearmanr*, and ordinary least squares regression was performed on log-transformed variables with *statsmodels.formula.api*’s *ols* function.

### Antigen-specificity prediction

We predicted Ebolavirus-specificity of EBL2001 participant V_H_ sequences via shared IGHV and amino acid identity over length-matched CDRH3. In the main text, we used a 70% CDRH3 amino acid identity threshold but explore 80% or 90% CDRH3 identity thresholds in Supplementary Materials. Using clonal relatives of known antibodies to predict antigen specificity is a common approach and was validated previously in a transgenic model (48). This method was implemented in Python and is published as a Python package, *clone_search_ab*.

## Results

### Compilation of a database of anti-EBOV antibody and nanobody sequences

**Figure 1:**
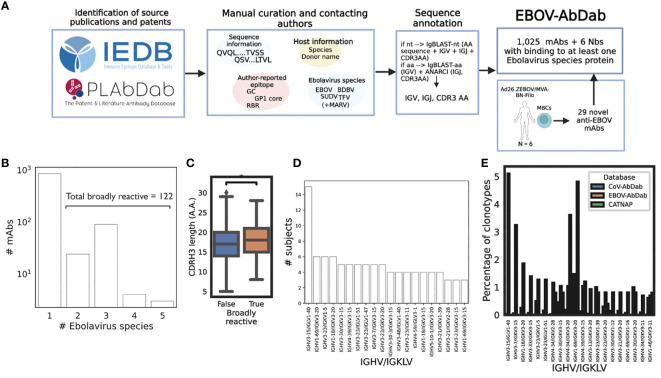
Curation of publicly-available Ebolavirus antibody sequences reveals common gene combinations. We manually curated a database of Ebolavirus-binding mAbs (N = 1,025) and Nbs (nanobodies, N = 6) from the workflow described in panel **(A)** Curated and annotated sequence information from the literature with labels such as viral species, protein and epitope, were combined with 29 novel mAbs derived from six Ad26.ZEBOV/MVA-BN-Filo vaccines post-dose 2 to produce a comprehensive database. We curated viral species **(B)**; among the human subset of the data, we identified 122 entries which displayed binding to more than one Ebolavirus. While we are careful to compare these broadly reactive mAbs with mAbs which only have one Ebolavirus label as the absence of data is not equivalent to negative data, we noted a significantly longer CDRH3 length in the broadly reactive subset (p = 0.03) **(C)**. We then analyzed the database to identify public antibodies with respect to IGHV/IGKLV gene pairings **(D)** and noted exceptional publicity of the IGHV3–15/IGLV1–40 lineage of antibodies. To put these frequencies into the context of independent viral antibody databases, we compared clonotype frequency within the database to CoV-AbDab and CATNAP (a database of HIV antibodies), and note that IGHV3–15/IGLV1–40 antibodies are rare in other anti-viral antibodies being 174x more common in EBOV-AbDab than CoV-AbDab, and not observed among HIV antibodies **(E)**.

To collate current knowledge on antibodies against Ebolavirus antigens, we compiled a reference database of antibodies with known specificity for Ebolavirus proteins, collected from academic publications and patents which were identified using the Immune Epitope Database (IEDB) and the Patent/Literature Antibody Database (PLAbDab) (34, 49). In addition, we included the sequences of 29 previously unpublished mAbs (Rijal et al, *in prep*). These novel mAbs were generated from plasmablasts sorted on EBOV-GP from six participants in the same trial who had received an Ad26.ZEBOV dose 1 and a MVA-BN-Filo Ebola dose 2. Altogether, this resulted in a database of 1,019 antibodies and 6 nanobodies, with the encoding IGHV or IGKLV gene and CDRH3 or CDRL3 sequences provided as a minimum. The workflow for our database curation can be seen in **Figure 1A**.

The majority of the antibodies and nanobodies (939/1,025) targeted the glycoprotein (GP), with 13 targeting the nucleoprotein (NP), 11 targeting the matrix protein VP40, and a single antibody each targeting VP35 and VP30. The majority of the database was of human origin (981/1,025) with the remainder of antibodies of murine (36) and macaque (2) origin and all nanobodies derived from llamas (6).

One of the information fields we collected was the Ebolavirus species known to be targeted by each mAb (EBOV, TFV, BDBV, SUDV, as well as non-Ebolavirus MARV). We curated this information if it was available, but do not distinguish absence vs. negative (i.e., if a mAb is labeled as “EBOV”, this does not mean that it does not bind to BDBV, simply that this has not been observed). Focusing on the human subset of the data, we identified 122 entries which bind to at least two Ebolaviruses, which we refer to as broadly reactive (**Figure 1B**). We are careful not to draw too firm conclusions with respect to this label, however we do note that the average CDRH3 length among clonotypes within this “broadly reactive” category is significantly greater than in antibodies with confirmed binding to a single species (p = 0.03, Mann-Whitney U-test) (**Figure 1C**).

As we are beginning to understand the role of particular IGV genes in determining immunodominance, and since much of the Ebolavirus mAb literature is understood within the context of these genes, e.g. IGHV1–69 and the mucin-like domain (MLD) or IGHV3–15/IGLV1–40 mAbs and the RBR (25, 50, 51) we analyzed our database with respect to these IGV gene pairings. As we collected author-reported donor labels (e.g., EVD5 or Subject 45), we looked at how many donors each gene pairing was identified in. IGHV3–15/IGLV1–40 mAbs were discovered in fifteen participants with the next most public pairing being observed in six participants (IGHV1–69/IGKV3–20, IGHV3–23/IGKV1–5 and IGHV1–18/IGKV3–20) (**Figure 1D**). We then calculated the frequency of these pairings based on unique clonotypes (IGHV/IGKLV and 90% amino acid identity in the CDRH3) and compared this frequency to that observed in a much larger, independent viral antibody database (CoV-AbDab) (**Figure 1E**). While IGHV3–15/IGLV1–40 mAbs constitute around 5% of clonotypes within EBOV-AbDab, they constitute just 0.03% of CoV-AbDab, i.e. are 174x more frequent in EBOV-AbDab than CoV-AbDab. There are a further 55 IGHV/IGKLV gene pairings which are at least 10x more frequent in EBOV-AbDab than CoV-AbDab. The differential frequency of IGHV3–15/IGLV1–40 mAbs is primarily driven by the frequency of IGHV3–15 (being 4.9x more frequent vs IGLV1–40 being 1.2 more frequent).

### A novel lineage of IGHV3–15/IGLV1–40 mAbs and rediscovery of a known one

**Figure 2:**
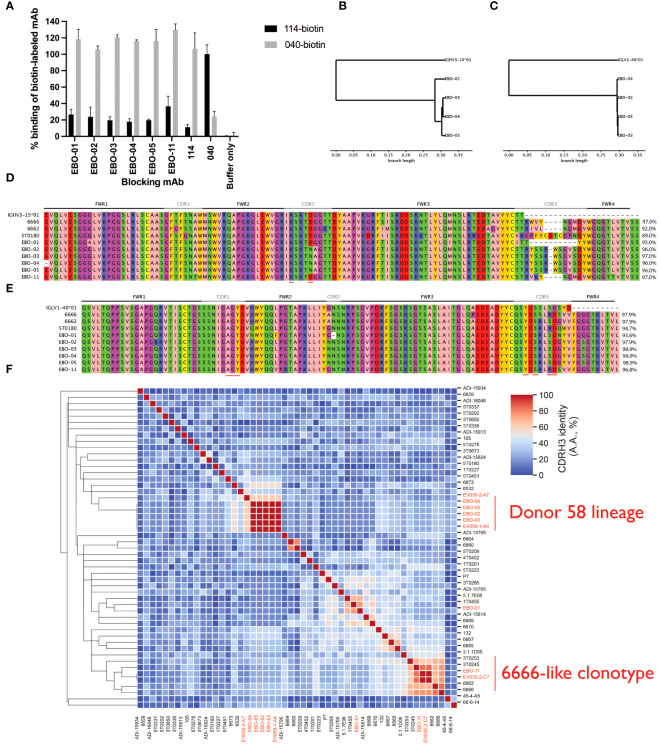
Novel IGHV3–15/IGLV1–40 mAbs fall into two groups according to their CDRH3s, the Donor 58 lineage and 6666-like clonotype eight IGHV3–15 mAbs were recovered from two subjects, five from a single subject. We tested six mAbs, EBO-01 to EBO-05, from one subject, and EBO-11 from the other subject, for competition with mAb114, which binds the RBR, and mAb040 which binds a non-overlapping epitope on the glycan cap, on EBOV GP. All six IGHV4-15 mAbs competed with mAb114 suggesting an epitope on the RBR **(A)**. EBO-02 to EBO-05 likely derived from the same clonal expansion: UPGMA dendrograms calculated based on the nucleotide sequences are shown for the VH **(B)** and VL **(C)** with the originating IGHV3–15*01 and IGLV1–40*01 as the outgroup. Panels **(D, E)** show the IMGT-gapped amino acid sequence alignments; red bars on the bottom indicate the paratope residues which are conserved across all three structures solved by Cohen-Dvashi et al. (2020). 5T0180, one of these mAbs, is also included, as are 6666 and 6662 which are RBR-binding mAbs discovered in ChAd.ZEBOV/MVA-BN-Filo vaccinees. Amino acid identity across the IGHV-encoded region is displayed. Germline D61 (IMGT) in CDRH2 which is reported to be a paratope residue is substituted for asparagine in EBO-02, -03 and -04 mAbs while K57 is retained. The new lineage lacks the S113R substitution observed in 5T0180, 6666 and EBO-11. We examined the CDRH3s of our IGHV3–15/IGLV1–40 mAbs within the context of all IGHV3–15/IGLV1–40 mAbs within EBOV-AbDab, with the novel mAbs highlighted in red **(F)**. One subset of our novel mAbs represent one subcluster with 100% CDRH3 identity and maximally 55% CDRH3 identity to any previously described mAb (Donor 58 lineage). We identify a separate subcluster which we refer to as the 6666-like lineage (as 6666’s CDRH3 is central) with greater CDRH3 homology.

We generated 29 novel mAbs from memory B cells of Ad26.ZEBOV/MVA-BN-Filo vaccinees. We noted the frequency of IGHV3–15/IGLV1–40 mAbs (eight mAbs in four clonotypes, two clonotypes in each donor). We examined two lineages of IGHV3–15/IGLV1–40 antibodies from one donor (Donor 58; EBO-1 and EBO2–5) and a single mAb from Donor 35 (EBO11). We measured the competition of these mAbs with mAbs114 (RBR) and mAb040 (GC). These mAbs competed for binding to EBOV GP with mAb114 making it probable that all three lineages target the RBR (**Figure 2A**). The EBO2–5 lineage is visualized via dendrograms in **Figure 2B** (VH) and **Figure 2C** (VL). All six tested mAbs are shown aligned via IMGT numbering in **Figure 2D**, alongside two separate independent IGHV3–15/IGLV1–40 mAb lineages from the literature - 6666 and 6662 derived from ChAdOx.ZEBOV/MVA-BN-Filo vaccinees and 5T0180 derived from rVSV vaccinees (31, 32). As described by Cohen-Dvashi and colleagues, there is evidence of relative conservation of germline-encoded paratope residues in both the VH and VL, but significant diversity in the CDRH3 (50).

We wanted to contextualize our novel mAbs within our broader database of IGHV3–15/IGLV1–40 antibodies. Given the conservation of the CDRL3, we focused on the non-conserved CDRH3 (**Figure 2F**). Hierarchical clustering of non-length matched CDRH3 amino acid identity reveals two subclusters: one is the lineage we describe here (represented by EBO-2–5) which has maximally 62% CDRH3 identity to any previously described IGHV3–15/IGLV1–40 mAb thus representing a novel lineage which we refer to as the Donor 58 lineage. The second subcluster shows CDRH3 homology to mAbs isolated from rVSV vaccinees (3T0245 and to a lesser extent 3T0253) and ChAdOx.ZEBOV/MVA-BN-Filo vaccinees (6662 and 6666). We refer to this as the *6666-like clonotype* (as the 6666 CDRH3 is the central CDRH3 in terms of sequence identity).

### BCR repertoire sequencing suggests the proliferation of B cells carrying non-mutated IgG BCRs following the monovalent dose 1, with evidence for increasingly mutated BCRs with increasing dose 1-dose 2 interval

When B cells are activated by an antigen stimulus, AID is switched on and the B cell undergoes class switching from IgM/IgD to IgG, A and E and B cell clones responding to the antigen stimulus will accumulate mutations in the variable region of the BCR as the evolve to have higher affinity binding for their epitope. Furthermore, B cells that take on the plasmablast (PB) phenotype rapidly proliferate in a process known as clonal expansion. Finding clonally-related, class-switched or mutated BCR sequences is indicative of antigen exposure.

**Figure 3:**
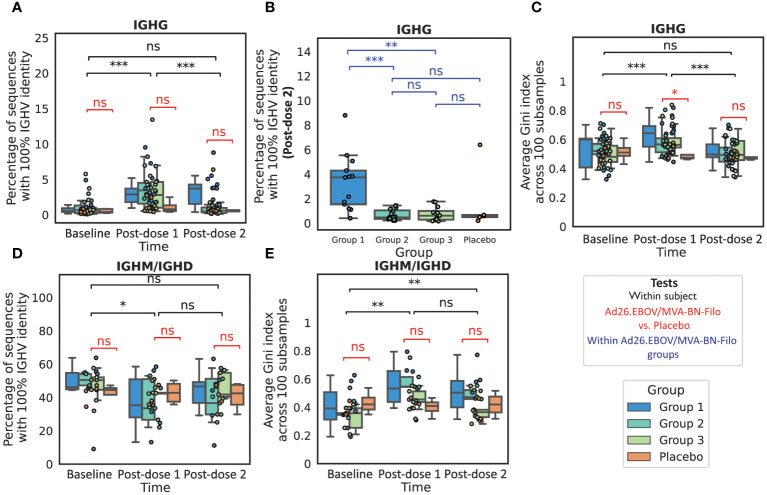
The IgG and IgM repertoires exhibit features of antigen exposure following vaccination We noted a significant increase in the proportion of the sequenced IgG repertoire that was non-mutated, defined over the IGHV region **(A)**, post-dose 1 relative to baseline, resulting in a significantly higher proportion of non-mutated sequences in the vaccinees than the placebo group. There was no significant increase post-dose 2 when grouping all boost interval cohorts, however we found that the proportion of the repertoire that was non-mutated was significantly higher in Group 1, which had the shortest dose 1-dose 2 interval of 4 weeks, than in Group 2 (8 weeks), Group 3 (12 weeks) or the Placebo group **(B)**. We next looked at the repertoire polarity in terms of the Gini index (higher Gini indices reflect increased polarization) averaged over 100 subsamples to the minimal number of sequences in the comparison, and noted a significant increase in Gini index from baseline to post-dose 1 in the IgG repertoires followed by a significant decline post-dose 2 to comparable polarization as observed at baseline **(C)**. In IGHM repertoires (with a reduced cohort of 21 subjects with 17 vaccines and four placebo), we noted a small but significant decrease in the proportion of the non-mutated repertoire post-dose 1, however the values were not significantly lower than observed in the placebo group **(D)**. The Gini index was significantly higher than at baseline in the IgM repertoires at both time points **(E)**, however the values observed in the vaccinee repertoires were again not significantly elevated in comparison to the Placebo group. (*, **, ***, ns: significant at the 5%, 1% and 0.01% level, and p ≥ 0.05).

Following dose 1, we noted a significant increase in the proportion of the IgG repertoire that was unmutated from an average of 1.0 ± 0.4% at baseline to 3.5 ± 0.9% post-prime in the non-placebo group (p<< 0.001, Wilcoxon test) (**Figure 3A**) suggesting an increase in frequency of class-switched but non-affinity matured BCRs. Following dose 2, the Group 1 participants still had elevated non-mutated BCRs (3.3 ± 1.4%) relative to both the placebo and Group 2 and Group 3 participants (0.7 ± 0.2%) at this time point (p = 0.001 and 0.002 for Group 1 vs. Group 2 and 3 respectively). For the Group 2 and Group 3 participants, the proportion of the repertoire that was non-mutated decreased to comparable levels to baseline and the placebo group (average 1.6 ± 0.6% in the non-placebo, vs. 1.7 ± 3.8% in the placebo) (**Figure 3B**). Group 1 had the shortest interval regimen of 4 weeks (vs. 8 and 12 weeks respectively). In the longer interval groups, the significant reduction in the proportion of the IgG repertoire that is non-mutated relative to post-dose 1 for Groups 2 and 3 is consistent with circulating B cells being generated from memory B cells (MBCs) that have had longer to undergo the process of affinity maturation and selection within the germinal center (>8 weeks for groups 2/3 versus 4 weeks for group 1).

To focus on the somatically-mutated, responding clonotypes that were likely to have undergone clonal expansion, we examined the 100 largest, somatically-mutated clonotypes in each repertoire. Measuring IGHV identity (percentage identity to the assigned IGHV allele) in this mutated subset provides a separate insight into how mutated an average responding BCR is, vs. the total repertoire. There was a significant increase in the median IGHV identity for the Groups 1–3 combined after both dose 1 and dose 2 (p<< 0.001) compared to baseline, with significantly higher median IGHV identities than the placebo at these time points (p = 0.003 and 0.003 respectively) (**Supplementary Figure 1A**). There was no significant difference in median IGHV identity for the 100 largest clonotypes *between* post-dose 1 and post-dose 2 with an average IGHV identity of 94.4 ± 0.7% and 94.6 ± 0.7% respectively (p = 0.72). There was a small but significant difference (p = 0.04) between Group 1 and Group 2 in the average IGHV identity in the 100 largest clonotypes post-boost, with Group 1 having a slightly higher average IGHV identity (95.2 ± 1.7%, vs. 94.5 ± 1.2% in Group 2) (**Supplementary Figure 1B**). In summary, these results suggest a post-dose 1 repertoire dominated by recently generated B cells with low or absent SHM. The total post-dose 2 repertoire has a comparable frequency of predicted memory BCRs to baseline, but with lower median IGHV identity; Group 1, with the shortest boost interval, has significantly more non-mutated sequences post-boost than Group 2 or Group 3, and mutated sequences tend to have slightly higher IGHV identity, suggesting that boost interval affects the nature of the B cell memory recall.

We next assessed repertoire polarity via the Gini index which is the area under the curve relating rank and cumulative abundance, averaged over 100 subsamples to the minimum repertoire size in the comparison (**Figures 3C, E**). In the IgG repertoires, we noted that while there was a significant increase in Gini index (repertoire polarity) from baseline to post-dose 1 (from 0.52 ± 0.03 to 0.60 ± 0.03), there was a significant decrease from post-dose 1 to post-dose 2 (0.51 ± 0.03, p<< 0.001) such that the expansion was comparable to baseline and the placebo at this time point (0.48 ± 0.03, p = 0.61). We would expect to find a comparable if not greater degree of clonal expansion post-dose 2 than post-dose 1, given that the post-dose 1 time point is at the tail end of the expected PB peak. We speculate that this could indicate a more polyclonal response engendered by the multivalent dose 2 than the monovalent dose 1.

In a subset of our cohort (N = 21), we performed IgM/IgD sequencing in addition to IgG sequencing. This is intended to provide a window into the naive repertoire, which is the non-mutated subset of the IgM/IgD repertoire, as well as IgM memory. We noted a small but significant reduction in the naive repertoire (non-mutated IgM/IgD) following dose 1, but not dose 2, from 26.3 ± 4.6% to 19.4 ± 4.6% (p = 0.01 and 0.91 respectively) (**Figure 3D**), however this was not significant relative to the placebo group (24.2 ± 15.3%) (p = 0.7). We noted a significant increase in the repertoire clonality from both baseline to post-dose 1 and to post-dose 2 (**Figure 3E**).

We looked into the longitudinal persistence of clones observed at baseline, post-dose 1 and post-dose 2. We first noted that on average the clonal overlap between baseline and post-vaccination repertoires was slightly lower than observed in the placebo group, but not significantly so. Focusing on the post-dose 2 repertoire, we found that there was a comparable proportion of clonotypes retained from post-dose 1 in the post-dose 2 repertoires of Group 1, 2 and 3 participants to one another and the placebo group. In the light of the higher abundance of non-mutated IgG sequences at post-dose 2 in Group 1, we specifically focused on the naive to mutated clonotype transition and found that while this appeared to be slightly greater in Group 1 than Group 2 or Group 3, the effect was not statistically significant. There was also no significant difference in the proportion of IgM clonotypes at a prior time point that were observed class-switched at the following time point.

On analyzing the relative proportions of isotype subtype frequencies among the IgG repertoires we found that the proportion of the repertoire occupied by IgG1 increased significantly post-dose 1, from a mean of 50.6 ± 4.2% to 64.8 ± 3.7%, and then again at post-dose 2 to 72.4 ± 3.4%. There were compensatory decreases in IgG2 (39.4 ± 4.3% to 24.0 ± 3.4% to 19.4 ± 2.8%) and IgG4 (1.6 ± 0.6% to 0.8 ± 0.3% to 0.6 ± 0.3%). The vast majority of clonotypes in this post-prime IgG1 increase were novel, in that they did not appear prior to vaccination (96.2 ± 1.1%). There were no significant changes in the isotype subtype frequencies in control participants.

We examined IGHV gene frequencies and while we noted several IGHV genes with significant changes in frequency throughout the course of vaccination among vaccinees in IgG and IgM repertoires, none of these changes were significant relative to those observed in the placebo group, post correction for multiple testing (**Supplementary Figures 2**, **3**). While the IGHV frequencies in IgM/IgD and IgG repertoires within participants at the same time point were reasonably well correlated, with average Spearman correlation coefficients of 0.95 ± 0.01, 0.94 ± 0.01 and 0.95 ± 0.01 at baseline, post-dose 1 and post-dose 2 respectively, the changes in IGHV genes observed in the IgG repertoire were not mirrored in the IgM repertoires (**Supplementary Figure 3B**). IgG and IgM repertoires were least correlated at post-prime, indicating divergence in the repertoires coincident with the aforementioned predicted PB peak (**Supplementary Figure 3C**).

These observations suggest an antigen-specific response in vaccinees both post-dose 1 and post-dose 2. While clonal expansion is a reliable marker for antigen-specificity, we decided to use computational immune repertoire mining to refine our prediction of the antigen-specific component of the response.

### A database method for the prediction of the EBOV GP-specific IgG repertoire

In order to predict the component of the repertoire that is likely to bind to one of the vaccine antigens, we used our database of Ebolavirus sequences to search for clonal relatives likely to share the same specificity. Clonal relatives were defined as sharing the same IGHV and 70% amino acid identity across the length-matched CDRH3. Predicted Ebolavirus-binding heavy chain sequences are referred to as “Ebolavirus hit sequences’’. As a control, we compared these results to clonotype predictions using a non-Ebolavirus antibody database built from the non-Ebolavirus-specific, human subset of the Immune Epitope Database (IEDB), as well as the human subset of a separate Coronavirus database (CoV-AbDab). The trial occurred prior to the COVID-19 pandemic, so there is not expected to be any systematic increase in the hit rate to the Coronavirus database. Furthermore, the vaccinees are UK-based and all lacked any IgG titer to the Ebolavirus GP antigen at baseline, therefore there is not expected to be an appreciable hit rate to the Ebolavirus antibody database at baseline.

**Figure 4:**
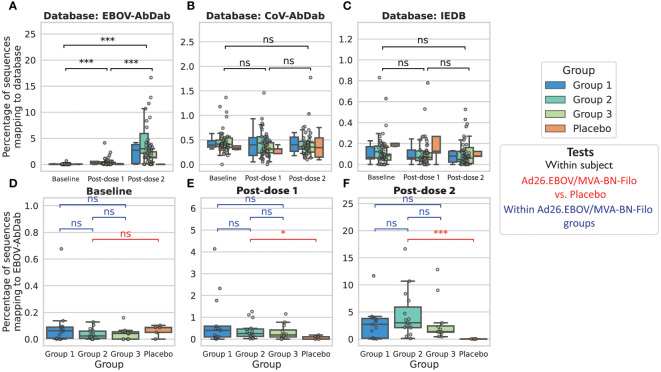
Predicted Ebolavirus-specific antibody sequences significantly increase in frequency post-dose 1 and post-dose 2 from baseline, while predicted Coronavirus and other antigen-specific sequences do not significantly change in frequency We used either our curated Ebolavirus antibody database, EBOV-AbDab **(A)**, a Coronavirus-specific antibody database (CoV-AbDab) **(B)** or a non-Ebolavirus database of antibodies to diverse antigens (IEDB) **(C)** to predict the subset of the IgG repertoire that is specific to an antigen in question, and found a significant increase in the percentage of sequences mapping to EBOV-AbDab (referred to as “hits”) throughout the course of vaccination, particularly post-dose 2, while there was no significant change in the percentage of sequences mapping to CoV-AbDab or the IEDB. This indicates that these are likely antigen-specific BCRs. For statistical testing, black bars show paired tests between time points **(A-C)**, while red bars show tests between vaccinees and the control group, and blue bars between groups of vaccinees **(D-F)**. There was no significant difference between the placebo group and Ad26.EBOV/MVA-BN-Filo vaccinees prior to vaccination (Mann-Whitney U-test; p = 0.25; red bar in panel **(D)**. Following dose 1, there was a significant increase in the proportion of EBOV-AbDab hits (p<< 0.001), and a further significant increase from post-dose 1 to post-dose 2 (p<< 0.001; black bars in panel **(A)**, resulting in significantly higher percentages of EBOV-AbDab hit sequences in the Ad26.EBOV/MVA-BN-Filo vaccinees vs. the placebo group post-dose 1 (p = 0.03) and post-dose 2 (p<< 0.001) (red bars, **(E, F)**. There were no significant differences between the different dose interval groups at any time point (blue bars, **(D-F)**. There were no significant differences in the hit rates to any database in the IgM/IgD repertoires (**Supplementary Figure 5**). (*, ***, ns: significant at the 5%, 1% and 0.01% level, and p ≥ 0.05).

We measured the proportion of sequences in the repertoire that were hits to the database, which is a function of both the number of hit clonotypes and their abundance. In line with our expectations, we observed a significant increase in the proportion of IgG Ebolavirus hit sequences in the vaccinees’ repertoires post-dose 2 (**Figure 4A**): at baseline, a mean of 0.06± 0.04% of sequences, and maximally 0.68%, were predicted to bind to Ebolavirus. There was a significant increase to 0.51 ± 0.25% post-dose 1 (maximally 4.1%) followed by a significant increase to 3.6 ± 1.2% (maximally 16.6%) post-dose 2. We did not observe any significant changes in the proportion of sequences predicted to bind to non-Ebolavirus antigens (**Figures 4B, C**). There was no signal in the placebo group, with a mean of 0.07 ± 0.05%, and 0.08 ± 0.11% and 0.03 ± 0.04%, and maximum of 0.10%, 0.18% and 0.06% of IgG sequences predicted to be EBOV-reactive, at baseline, post-dose 1 and post-dose 2 respectively (**Figure 4A**). There were no significant differences between the different dose 1-dose 2 interval groups in the proportion of the repertoire mapping to the database (**Figures 4D–F**). We note the same significance intervals, though with hit rates on average 3.6 or 36.0 times lower, using CDRH3 amino acid identity thresholds of 80% and 90% in addition to the 70% threshold used in the main figures (**Supplementary Figure 4**). We did not observe this signal in the IgM repertoires, with comparably very low hit rates and no significant difference in the proportion of IgM Ebolavirus hit sequences in the vaccinees repertoires post-dose 1 or post-dose 2 compared to baseline (**Supplementary Figure 5**).

We next looked at the diversity of these predicted hit sequences by looking at the originating clonotypes. We found a significant increase in the number of unique hit clonotypes post-dose 1 and post-dose 2 from on average 1.6 ± 0.6 at baseline to 6.1 ± 2.0 post-prime, and 23.3 ± 5.9 post-boost (**Supplementary Figure 6**). We found that 45% of clonotypes post-prime were also found within the same participant post-boost, i.e., these predicted antigen-specific sequences that arose during the first vaccination were also observed following the multivalent dose 2. By contrast, only 17.5% of hit clonotypes post-dose 2 were found at the preceding time point, i.e., the majority of post-dose 2 clonotypes were derived from lineages absent at the post-dose 1 time point in the same participant.

**Figure 5:**
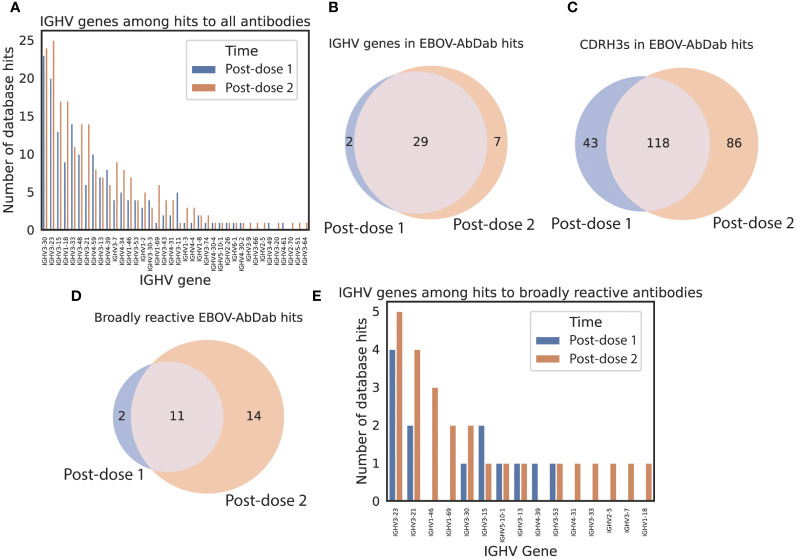
predicted hit sequences derive from diverse IGHV origins, and more predicted broadly reactive sequences appear following the second dose EBOV-AbDab hits derive from 38 IGHV genes **(A)**, the majority of which are seen at both time points **(B)**. There are 1.5x as many unique CDRH3s found among post-dose 2 hits than post-dose 1 hits **(C)**. We note that there are hits to twice as many broadly reactive mAbs post-boost than at post-dose 1, indicating that the dose interval may be conducive to developing broadly neutralizing mAbs **(D)**. The broadly neutralizing mAbs derive from 15 IGHV genes **(E)**.

To examine whether these novel clonotypes arose through somatic hypermutation of existing hit antibodies, we looked at the hits on the basis of IGHV origin (**Figure 5**). On average, hit sequences derived from 5.4 ± 0.9 different IGHV genes prior to vaccination, 7.3 ± 1.2 post-dose 1, and 10.1 ± 1.1 post-dose 2, revealing significant diversification in the genetic origins of the predicted antigen-specific component of the BCR repertoire within participants following the multivalent dose 2. Our sequences mapped to 166 mAbs within the EBOV-AbDab database (out of 981 human mAbs); these were encoded by 31 and 36 IGHV genes post-prime and post-boost respectively with the majority of IGHV genes observed at both time points (29).

Finally, we looked at the hits in the context of the breadth of reactivity to different Ebolavirus species in our database. Of 121 human mAbs with the “broadly reactive” label, there were hits to 27 in the post-dose 1 and post-dose 2 repertoires combined. There were hits to 13 mAbs post-dose 1 and 25 post-dose 2 of which 11 were shared (**Figure 5D**) indicating that the significant diversification of hit sequences observed post-dose 2 results in more sequences predicted to be broadly reactive appearing in the vaccinee repertoires.

### Predicted EBOV-specific sequences are found within expanded and public clones

Ebolavirus hit sequences post-dose 2 were on average found in larger clonotypes than the repertoire average: the mean size of a clonotype containing a hit sequence post-dose 2 had 73.8 ± 27.5 members, in contrast with the repertoire-wide mean of 18.6 ± 3.7. For 35 of 40 vaccinees, the mean clonotype size was larger for hit sequences than the repertoire-wide mean, while for 24 of 40 participants at least one of the ten largest clonotypes contained hit sequences (including 12 participants for which the largest clonotype mapped to the database): for ⅔ of our cohort of vaccinees, our database approach was sufficiently powerful to be able to map at least one of the ten most expanded IgG clonotypes post-dose 2 to characterized mAbs. In one participant, four of the ten largest clonotypes had a hit to our database. Our coverage of the vaccinees’ most expanded clonotypes post-dose 2 demonstrates the strength of database-based specificity prediction.

**Figure 6:**
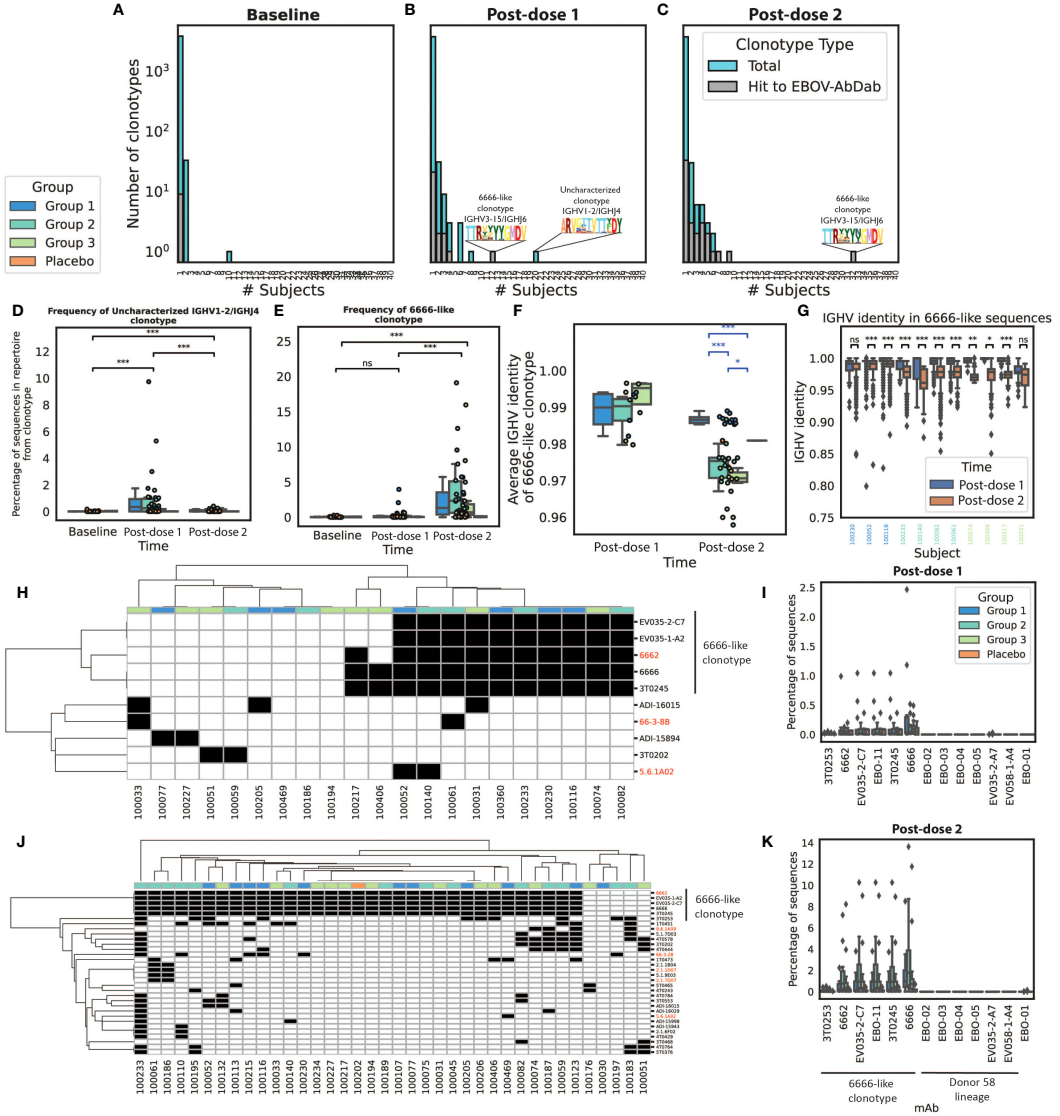
Many of the most public clonotypes are predicted to be antigen-specific by our method, with the most notable being the 6666-like lineage which increases in frequency and has reduced IGHV identity post-boost we explored convergence among the 100 largest clonotypes in each IgG repertoire. We noted limited convergence at baseline with the exception of an IGHV3–7 lineage found in ten subjects **(A)**. Post-dose 1, there were 50 clonotypes which were seen in at least two subjects of which six (20%) contained hits to EBOV-AbDab. Unfortunately, the most public clonotype observed in 20 subjects was not a hit to our database (referred to as the uncharacterized IGHV1–24/IGHJ4 clonotype), however the next most public clonotype was a 6666-like clonotype which we had already noted for its publicity within the database itself (**B**). Post-dose 2, there were also 50 public clonotypes, of which 14 (28%) were hits to the database; most notably, the 6666-like clonotype was observed within the 100 most abundant clonotypes of 32/40 vaccinees **(C)**. The uncharacterized IGHV1–24/IGHJ4 clonotype is not only public post-dose 1 but significantly increases in frequency, decreasing again post-dose 2 to comparable levels as at baseline **(D).** By contrast, the 6666-like clonotype significantly increases from baseline to post-dose 2 (p<< 0.001) but is not significantly increased in frequency post-dose 1 (p = 0.11) **(E)**. We focused on this 6666-like clonotype to look for evidence of somatic hypermutation. Interestingly, we found post-dose 2 that this clonotype was significantly less mutated in Group 1, with the shortest boost interval **(F)**. We looked at this lineage on a per-subject basis in the eleven subjects in which there were hit sequences at both post-vaccination timepoints; using a permutation test, we identified four subjects for which there was sufficient evidence that sequences were more mutated post-dose 2 **(G)**. Focusing further on the convergent hit clonotypes, it can be seen that at both post-dose 1 **(H)** and post-dose 2 **(J)** the 6666-like lineage is the most public hit clonotype (red labels correspond to broadly neutralizing antibodies; black boxes indicate presence of the lineage within the 100 largest clonotypes). Of the two lineages we noted in **Figure 2**, the 6666-like lineage is significantly higher frequency after both dose 1 **(I)** and dose 2 **(K)**, and more public than the Donor 58 lineage. (*, **, ***, ns: significant at the 5%, 1% and 0.01% level, and p ≥ 0.05).

These hit clonotypes were also exceptional with regards to their publicity (**Figure 6**). Focusing on the 100 largest clonotypes per subject at each time point, we noted 50 clonotypes that were found in more than one subject post-dose 1 or post-dose 2 (bars in blue). Of these 50 public clonotypes, 6 post-dose 1 and 14 post-dose 2 mapped back to our EBOV-specific database (bars in gray) (**Figures 6A–C**). Post-dose 1, the most public clonotype which was observed in 20 participants, was an IGHV1–2/IGHJ4 clonotype that did not match to our EBOV-AbDab database nor to any antibody in the IEDB or CoV-AbDab. This post-dose 1 clonotype significantly reduced in frequency post-dose 2 (**Figure 6D**). The second most public clonotype post-dose 1 was observed in 12 participants, corresponding to an IGHV3–15/IGHJ6 clonotype with hits to the 6666-like clonotype mAbs that we highlighted in EBOV-AbDab. This clonotype significantly increased in both publicity (**Figure 6C**) and within-participant frequency post-dose 2 (**Figure 6E**), being observed within the 100 largest clonotypes of 32 participants in our cohort of 40 vaccinees. Focusing on this lineage, we noted lower IGHV identities post-dose 2; interestingly, the Group 1 participants had significantly fewer mutations in this lineage (**Figure 6E**). Permutation test on a per-participant basis on the subset of subjects which had the lineage both post-dose 1 and post-dose 2, revealed a significant decrease in IGHV identity within the majority of participants (**Figure 6F**).

**Figure 6G** shows the presence/absence of each hit present in public (in top 100) clonotypes in each participant with any predicted hits post-dose 1; the most public clonotype is clearly the 6666-like clonotype, which is present at a greater frequency than the other set of mAbs we highlighted, our novel lineage discovered within Donor 58 (**Figure 6H**). **Figures 6J, K** show the same results post-dose 2, where the number of public clonotypes can be seen to be larger, with again the 6666-like clonotype standing out for its frequency and the number of participants in which it is observed.

### The proportion of predicted Ebolavirus-specific sequences correlates with fold-change in anti-EBOV IgG titer

**Figure 7:**
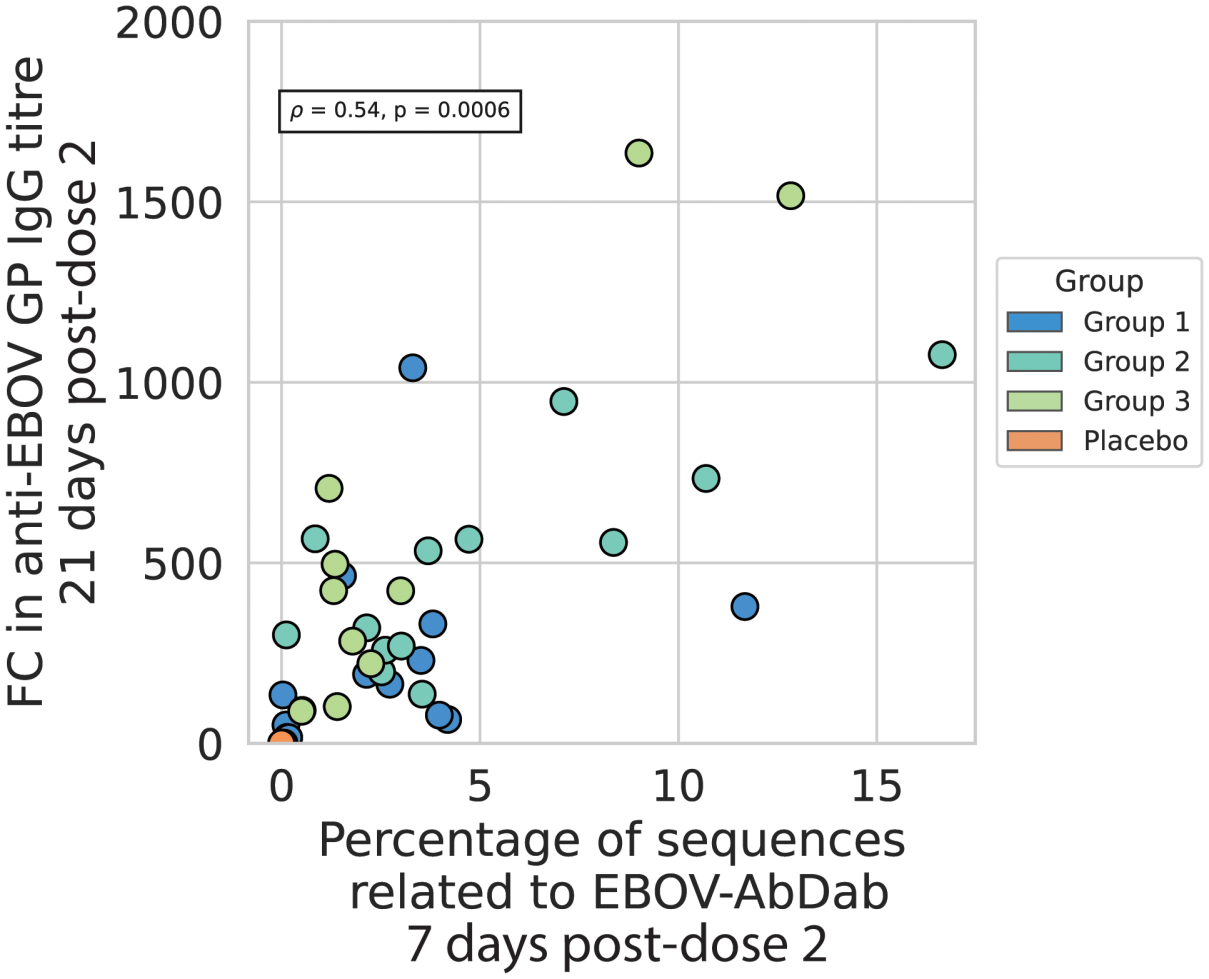
The percentage of sequences related to EBOV-AbDab post-dose 2 correlates positively with the fold change in anti-EBOV GP titer from baseline to 21 days post-dose 2 the percentage of sequences related to EBOV-AbDab is positively correlated with the fold-change in anti-EBOV GP IgG titer 21 days post-dose 2, with a Spearman’s correlation coefficient of 0.54, p<< 0.001. Given that the fold change is higher on average for Group 2 and Group 3 than Group 1, we added Group as another variable in an OLS regression on the log-transformed values; the total regression had an R^2^ of 0.51 with the hit rate variable positively associated with a correlation coefficient of 0.43 and p-value of 0.001. There was no significant correlation with hit rate post-dose 1 (**Supplementary Figure 7**).

We found a significant correlation between the proportion of Ebolavirus hit sequences in the repertoire 7 days post-boost and the anti-EBOV IgG titer 21 days post-dose 2, after adjusting for an established group effect (p = 0.001, R^2^ = 0.51) (**Figure 7**). The total Spearman’s rho coefficient, not accounting for the different groups, was 0.54 (p = 0.0006). There was no significant correlation with the proportion of Ebolavirus hit sequences at the post-dose 1 time point (p = 0.56) nor with the Gini index at either time point (p = 0.86 and 0.12 for post-dose 1 and post-dose 2 time points respectively) (**Supplementary Figure 7**).

## Discussion

Vaccine development is supported by improvements in our understanding of the humoral immune response to both natural infection and vaccination. This includes gaining insights into epitope immunodominance, the genetic composition of the BCRs targeting those epitopes, how vaccine-induced immunity may generalize to novel variants, and how particular populations respond differently (52). Repertoire sequencing’s utility in this context is its view of the repertoire in depth, particularly in the case of bulk VH sequencing where tens to hundreds of thousands of cells can be sequenced. A limitation, in comparison to the wealth of possible single-cell assays, is the loss of the native pairing information which would allow expression and testing of BCRs of interest, as well as the loss of transcriptional or cell surface marker information which would inform on B cell phenotype. Monoclonal antibodies from sorted cells provide information about antigen-specificity and functionality, but offer a limited window into the diversity of the immune response. Here, computational immune repertoire mining allowed us to somewhat combine the strengths of these two techniques. This database-based technique, validated in a transgenic model system in previous work, has been used in previous studies as validation of antigen-specificity of public clonotypes, for example in the study by Galson and colleagues in which the Coronavirus antibody database (CoV-AbDab) was used to provide evidence of antigen-specificity of convergent clonotypes (48, 53). With the ongoing expansion of available immune repertoire sequence data and monoclonal antibody discovery, we envisage that this approach will become increasingly useful.

In EBL2001 vaccinees we found repertoire polarization following dose 1 in both the IgG and IgM repertoires, a significant increase in the proportion of non-mutated IgG sequences and decrease in the proportion of non-mutated IgM sequences. Among the IgG repertoires, we noted a significant increase in the frequency of the IGHG1 subclass and compensatory decrease in the frequency of the IGHG2 and IGHG4 subclasses. The post-dose 1 B-cell repertoire signature is indicative of clonal expansion and class switching consistent with a plasmablast peak. There was a notable lack of these signatures following the MVA-BN-Filo dose 2, which is consistent with transcriptomic data in which genes related to B cell activation that are clearly upregulated seven days after the Ad26.ZEBOV dose 1 are not significantly upregulated (relative to baseline) following the MVA-BN-Filo dose 2 (54).

The most notable property of the post-dose 2 IgG repertoires was the significantly elevated proportion of sequences predicted to bind to the Ebolavirus glycoprotein according to our database method, which were found disproportionately in expanded and public clonotypes. The most exceptional publicity we observed was in the 6666-like lineage, which was within the 100 largest clonotype post-boost in 32 participants, and which we had already noted as the most public lineage of antibodies in our reference database. An increase in IGHV3–15 frequency was observed by BCR-seq in primary vaccination with ERVEBO by Erhardt and colleagues (32) as well as via RNA-seq by Blengio and colleagues (54). IGHV3–15 thus plays a clear role in the B cell response to Ebolavirus vaccination, from its abundance in monoclonals isolated from at least fifteen EVD survivors and vaccinees, to its appearance in bulk BCR-seq data in both our own Ad26.ZEBOV/MVA-BN-Filo cohort and Erhardt and colleague’s ERVEBO cohort, and finally in bulk RNA-seq data.

However, it is not clear that the role this class of antibody plays is equal in both infection and vaccination. Davis and colleagues performed bulk BCR sequencing in a number of EVD survivors and IGHV3–15 was not noteworthy; rather, IGHV3–13 was identified as appearing convergently in their monoclonals isolated from two EVD survivors (55). In Chen and colleagues’ 2023 study, IGHV1–69 and IGHV1–2 were the highest frequency among ~10,000 EBOV GP specific clonal lineages sequenced in a single EVD survivor (30). The simplest hypothesis for this discrepancy is that the EVD survivor B cells tend to be from the memory population and collected months post-infection, vs. the plasmablast sequences that we are most likely sampling eleven- and seven days post-prime and post-boost, and maximally 3 months after primary vaccination. The presence of IGHV3–15 among MBC-derived mAbs, as well as the reappearance of more somatically-mutated IGHV3–15 lineages post-boost, indicates that cells expressing this lineage of antibody do indeed enter the memory compartment.

There are further more complex differences among these studies that lie in the broader immunological context of vaccination vs. infection. There is clearly a major role for IGHV1–69 and IGHV1–2 in antiviral B-cell responses more generally to influenza, HIV and hepatitis C virus which could indicate that there is some induction method for these genes that is secondary in vaccination (56). Alternatively, this discrepancy could be immunogenetic. Our Ad26.ZEBOV/MVA-BN-Filo cohort is primarily Caucasian, while Ebolavirus is endemic to West Africa. The role of immunogenetics in the expressed repertoire is only now beginning to be understood due to historic difficulties in resolving the immunoglobulin locus at high-throughput (57–59).

We identified a correlation between the proportion of the repertoire that is predicted to bind to Ebolavirus post-boost and the anti-EBOV IgG titer, however we did not sequence the serum antibodies via Ab/Ig-seq to verify that there was any overlap with the sequences we predicted as antigen-specific. Some have reported little overlap between BCR-seq and the serum, and this was found to be true of the MBC repertoire and serum antibodyome in an Ebolavirus survivor (30, 60, 61). However, the repertoire and serum overlap should be sensitive to when the two experiments (cellular vs. serum) are performed, as well as to the immunological context – we identified a correlation between the BCR repertoire at seven (cellular) and 21 days (serum) post-boost, in the context of four protein antigens in a non-replication competent viral vector. We would not expect findings in the context of natural infection, with significantly longer or shorter intervals between the two experiments, to generalize to our own findings. Jackson and colleagues found a positive correlation between change in clonality index (comparable to Gini index) and fold-change in anti-HA titer following influenza vaccination (62). With a cohort of just five participants, Trück and colleagues identified a correlation between predicted Hib (*Haemophilis influenzae*)-specific CDR3 sequences and anti-Hib avidity index (63). Our cohort of 45 participants provides stronger statistical evidence that BCR repertoire features can correlate with IgG titer. Whether our predicted antigen-specific antibodies contribute to humoral immunity is a key question that could be addressed via Ab/Ig-seq.

Given the extremely large possible combinatorial diversity of the antibody response, it was surprising to us that existing Ebola-specific mAb sequences were sufficient to describe at least one of the ten most expanded clonotypes post-dose 2 in more than 2/3 of our cohort of vaccinees. Public clonotypes have been identified in BCR-seq data from diverse infection and vaccination contexts (influenza, dengue, HIV-1, coronavirus, Hepatitis C) (30, 62, 64–69) and appear to be the rule rather than the exception, mediated by common physicochemical motifs encoded by both the germline genes and shared somatic hypermutation (70, 71). We observed significantly more public clonotypes following the secondary immunization – we do not yet know whether this is a common feature of the BCR repertoire in primary vs. secondary exposure, or a result of the different viral vectors. Given the ubiquity of public clonotypes, BCR repertoire data should be understood within the context of previously described antibodies to antigens of interest. It would be interesting to consider how large a database of monoclonals would need to be to completely cover the most expanded clonotypes in a cohort of a given size. Continued antibody discovery efforts combined with standardization and deposition of antibody sequence and epitope data in public databases will be critical to the success of these methods.

## Data availability statement

The original contributions presented in the study are publicly available. This data can be found here: https://zenodo.org/records/10631605. The original codes presented in the study are found here: https://github.com/erichardson97/CloneSearch.

## Ethics statement

The studies involving humans were approved by the French national Ethics Committee (CPP Ile de France III; 3287), the French Medicine Agency (150646A-61), the UK Medicines and Healthcare Products Regulatory Agency (MHRA), and the UK National Research Ethics Service (South Central, Oxford; A 15/SC/0211). The study was done according to the current Declaration of Helsinki and the Good Clinical Practice guidelines. The studies were conducted in accordance with the local legislation and institutional requirements. The participants provided their written informed consent to participate in this study.

## Author contributions

ER: Conceptualization, Data curation, Formal analysis, Methodology, Software, Visualization, Writing – original draft, Writing – review & editing. SB: Conceptualization, Data curation, Formal analysis, Methodology, Investigation, Project administration, Writing – original draft, Writing – review & editing. FM: Writing – review & editing. LS: Data curation, Formal analysis, Methodology, Investigation, Writing – original draft, Writing – review & editing. PR: Data curation, Formal analysis, Methodology, Investigation, Writing – original draft, Writing – review & editing. MG: Data curation, Investigation, Writing – review & editing. VV: Data curation, Investigation, Writing – review & editing. JT: Data curation, Investigation, Writing – review & editing. EC: Data curation, Investigation, Methodology, Project administration, Writing – review & editing. DO'C: Investigation, Conceptualization, Writing – review & editing. KL: Writing – review & editing. AT: Writing – review & editing. BP: Writing – review & editing. AP: Writing – review & editing, Funding acquisition, Supervision. CD: Conceptualization, Writing – original draft, Writing – review & editing, Supervision, Funding acquisition. DK: Conceptualization, Writing – original draft, Writing – review & editing, Supervision, Funding acquisition.
