# Supplementary material for: Computational mining of B cell receptor repertoires reveals antigen-specific and convergent responses to Ebola vaccination
Source: Front Immunol. 2024 Jul 8;15:1383753. doi: 10.3389/fimmu.2024.1383753 (PMC11260629; doi:10.3389/fimmu.2024.1383753)
Supplement: Supplementary file 1 [file DataSheet_1.pdf]

Computational mining of B cell receptor repertoires reveals antigen-specific and convergent responses to Ebola vaccination
Supplementary Material

Eve Richardson[1,2,5], Sagida Bibi[2], Florence McClean[2], Lisa Schimanski[3], Pramila Rijal[3], Marie Ghraichy[4], Valentin von Niederhäusern[4], Johannes Trück[4], Elizabeth A. Clutterbuck[2], Daniel O'Connor[2], Kerstin Luhn[6], Alain Townsend[3], Bjoern Peters[5], Andrew J. Pollard[2], Charlotte M. Deane[1], Dominic F. Kelly*[2,7]

[1] Department of Statistics, University of Oxford, Oxford, U.K.

[2] Oxford Vaccine Group, Department of Paediatrics, University of Oxford, Oxford, U.K.

[3] Weatherall Institute for Molecular Medicine, University of Oxford, Oxford, U.K.

[4] Divisions of Allergy and Immunology, University Children's Hospital and Children's Research Center, University of Zurich (UZH), Zurich, Switzerland

[5] La Jolla Institute for Immunology, La Jolla, San Diego, USA

[6] Janssen Vaccines and Prevention, Leiden, Netherlands

[7] NIHR Oxford Biomedical Research Centre, Oxford University Hospitals NHS Foundation Trust, Oxford, U.K.

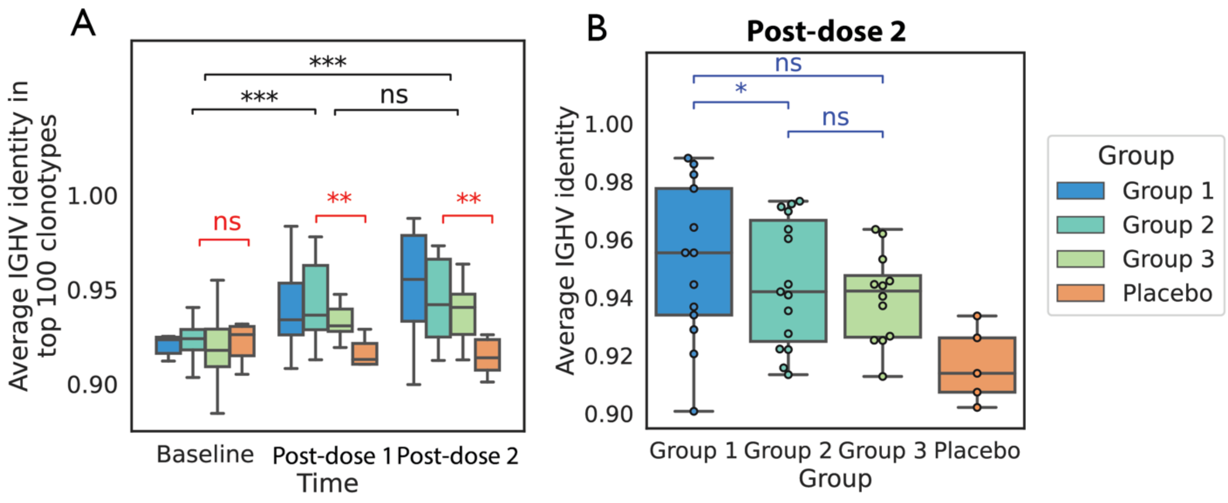

Supplementary Figure 1: the median IGHV identity in the 100 largest mutated clonotypes is significantly higher post-dose 1 and post-dose 2 than at baseline (**A**), and significantly higher in the vaccinees than the placebo (red bars). At the post-dose 2 time point, we note that the median IGHV identity is higher in Group 1 than Group 2 or Group 3, significantly so in the Group 1 vs. Group 2 comparison (p = 0.03).

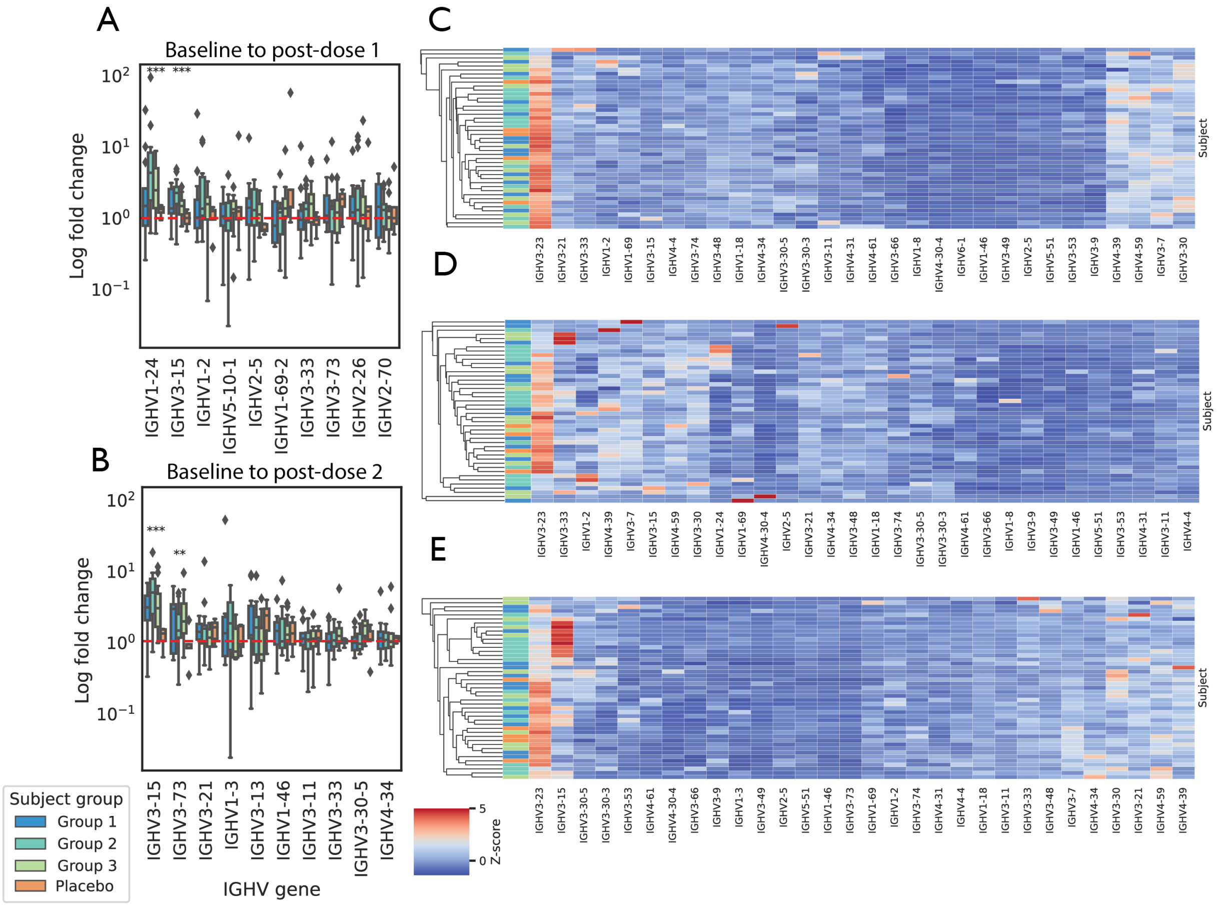

Supplementary Figure 2: the ten IGHV genes with the largest fold change from baseline to post-dose 1 (**A**) or post-dose 2 (**B**) are visualized, with asterisks indicating a significant increase in fold change at the relevant time point (Wilcoxon rank-sum test). Panels **C, D** and **E** show heatmaps with Z-normalized frequency of the thirty most abundant IGHV genes (for graphical purposes) at baseline (**C**), post-dose 1 (**D**) and post-dose 2 (**E**); IGHV genes and subjects are ordered according to a hierarchical clustering. Post-dose 1, IGHV1-24 and IGHV3-15 significantly increase in frequency; IGHV3-15 significantly increases further from post-dose 1 to post-dose 2, being the most highly expressed IGHV gene in ten participants (vs. one participant at baseline). We identified eight IGHV genes for which there was a significant difference at the 5% level between the study timepoints (repeated measures ANOVA; Benjamini-Hochberg FDR correction). Post-hoc Wilcoxon tests identified that from baseline to post-dose 1, there was a significant increase in the frequency of IGHV1-24 (an average FC of 7.0±5.0, $p \ll .001$), IGHV3-15 (1.9±0.3, $p \ll .001$) and IGHV3-64 (1.5±1.1, $p = 0.02$) and a significant decrease in IGHV3-23 (0.8±0.1, $p \ll 0.001$) and IGHV3-72 (0.8±0.3, $p = 0.02$). From baseline to post-dose 2, there was a significant increase in the frequency of IGHV3-15 (4.3±1.1, $p \ll .001$) and IGHV3-73 (2.4±0.6, $p = 0.003$), and a significant decrease in the frequency of IGHV3-23 (0.9±0.1, $p = .002$) and IGHV3-72 (0.9±0.3, $p = .003$). From post-dose 1 to post-dose 2, there was a significant increase in the frequency of IGHV1-2 (1.1±0.8, $p = 0.01$), IGHV3-15 (2.5±0.6, $p \ll 0.001$) and a significant reduction in the frequency of IGHV1-24 (0.5±0.3, $p \ll 0.001$).

However, none of these fold changes were significantly different from those observed in the placebo cohort, post-correction for multiple testing (Mann-Whitney U-test), with only IGHV3-15 post-prime and post-boost, and IGHV3-53 post-prime, significant prior to correction.

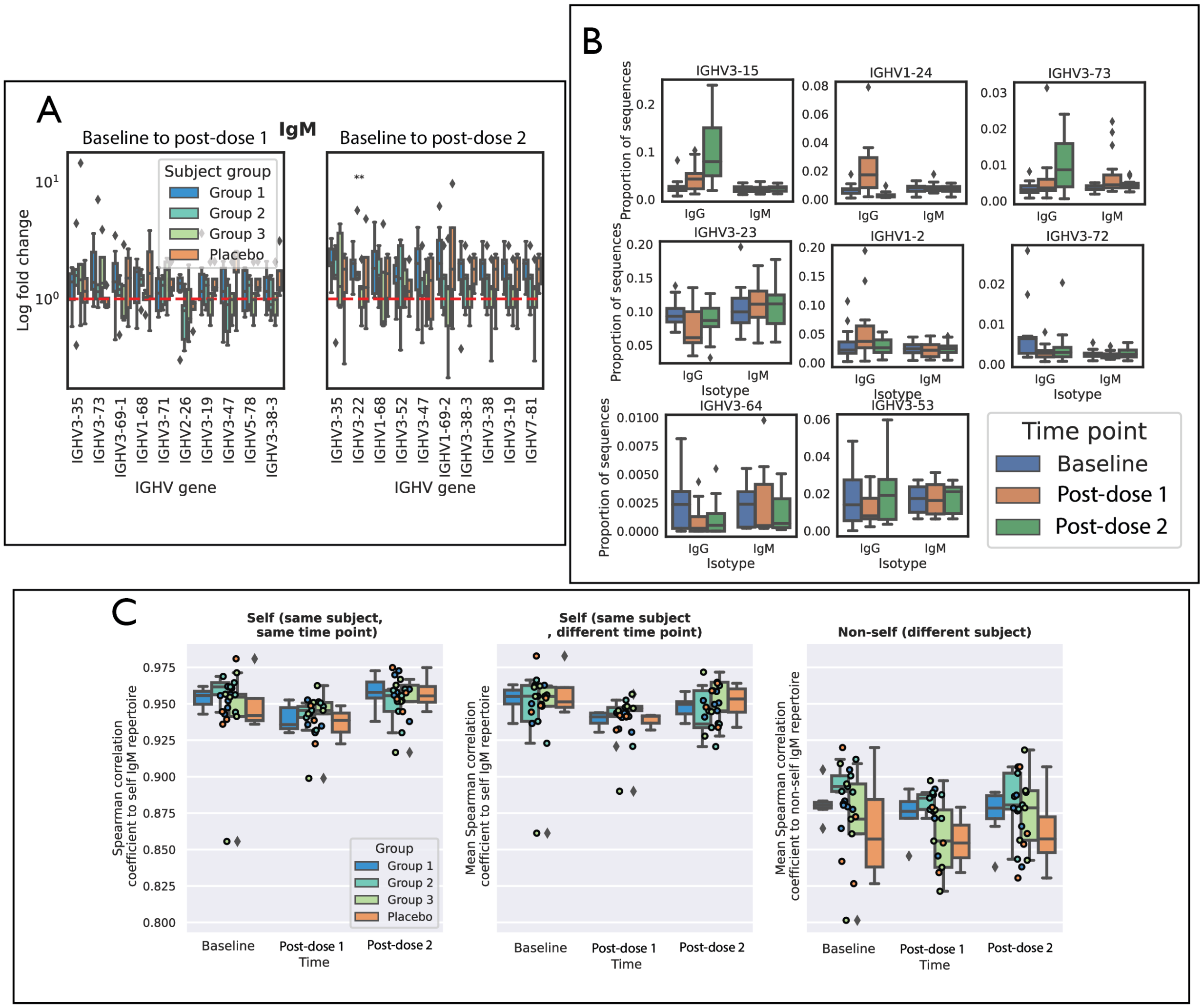

Supplementary Figure 3: log fold changes for the IGHV genes in the subset of IgM/IgD repertoires are shown; in no instance is the observed fold change significantly higher in the vaccinees than the control group (**A**). None of the most significantly changing IGHV genes in the IgG repertoires show the same pattern in the IgM repertoires (**B**), possibly because the IgG and IgM repertoires appear to diverge post-prime; while the Spearman correlation coefficient of IGHV gene usage is largely higher between IgG/IgM repertoires from the same subject and time point, correlation is lower post-prime than at baseline or post-prime (**C**).

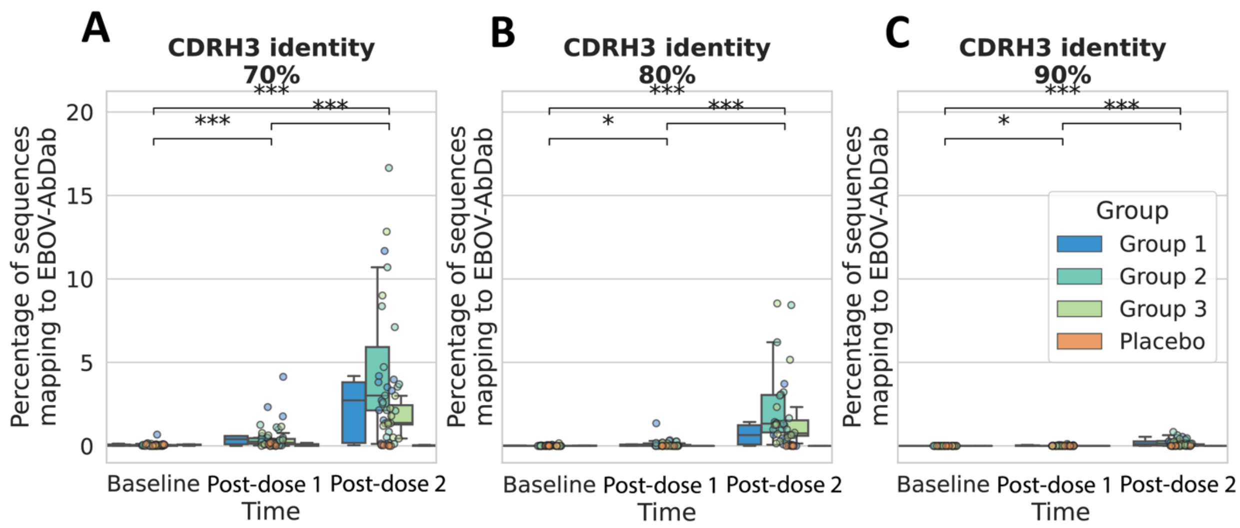

Supplementary Figure 4: at each CDRH3 threshold, there is a significant increase the percentage of sequences in the repertoire which map to EBOV-AbDab, though increasing the threshold significantly decreases the average hit rate by 3.6 and 36-fold going from 70% to 80%, and 70% to 90%, respectively.

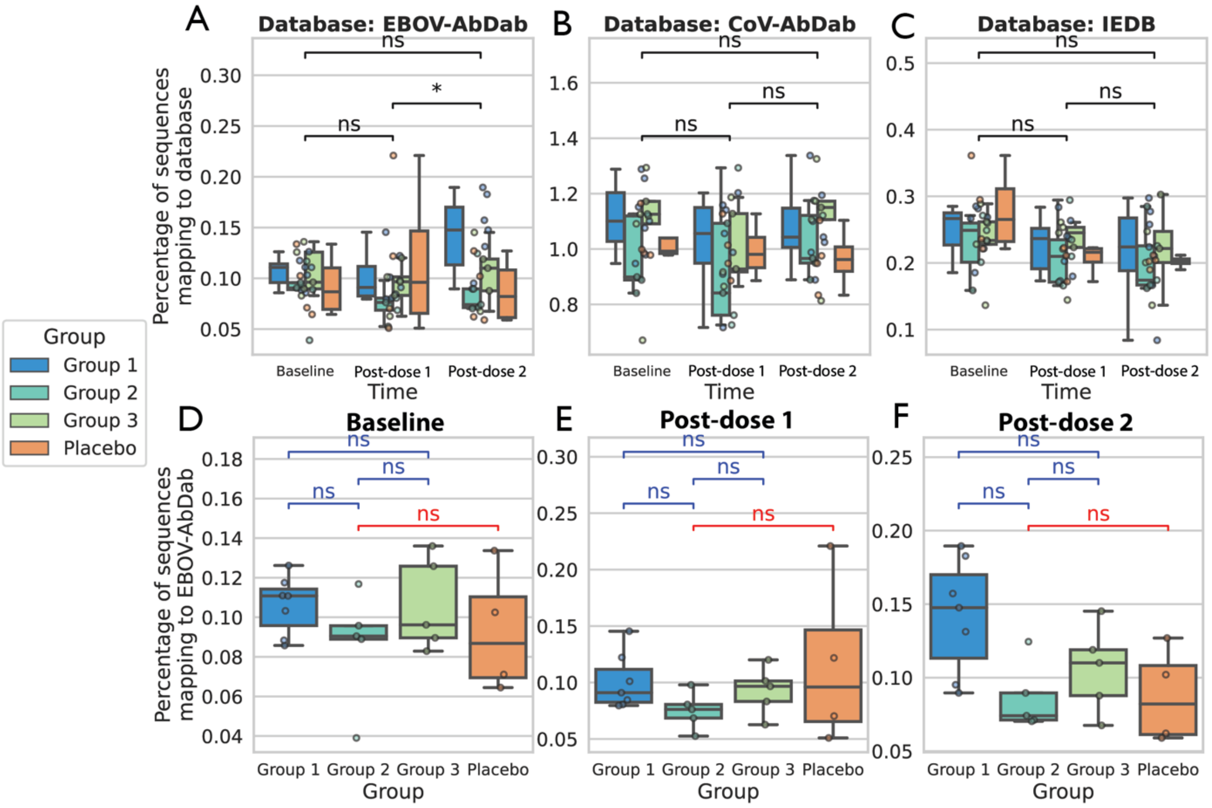

Supplementary Figure 5: we noted that the percentage of IgM sequences mapping to EBOV-AbDab was very low in comparison to the IgG repertoires with maximally 0.22% hit sequences in the IgM repertoires (in the placebo group) vs. 16.6% in the IgG repertoires. There were significantly fewer hits to EBOV-AbDab than to the IEDB ($p << 0.001$) or CoV-AbDab ($p << 0.001$). There was a significant increase in the proportion of hits in the post-dose 2 repertoire from 0.16±0.05 to 0.18±0.05% ($p = 0.03$, Wilcoxon Rank sum test) though the corresponding hit rate in the post-dose 2 repertoires was not significantly different from the Placebo group ($p = 0.2$) with average hit rates of 0.18±0.05% and 0.16±0.1% respectively.

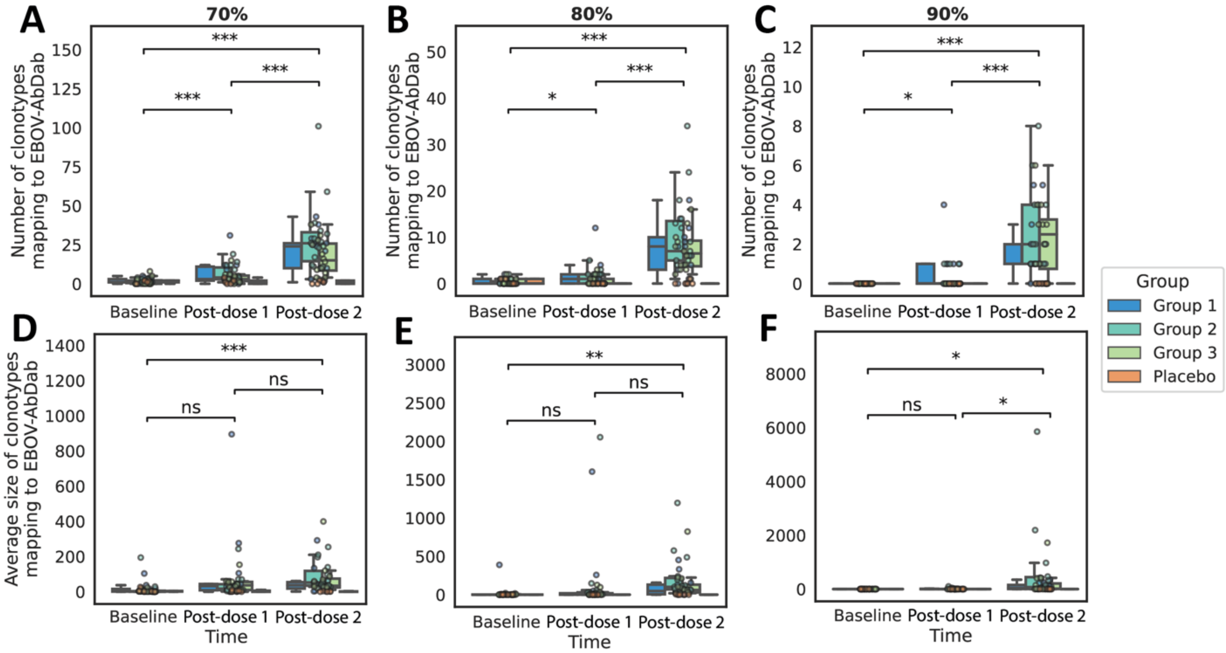

Supplementary Figure 6: database hit rate is a function of the number of hit clonotypes (top row) and the size of these clonotypes (bottom row). At each CDRH3 identity threshold, there is a significant increase in the number of clonotypes that are hits to the EBOV-AbDab database (**A, B, C**). The average size of these hit clonotypes is significantly larger post-dose 2 for each selected threshold (**D, E, F**).

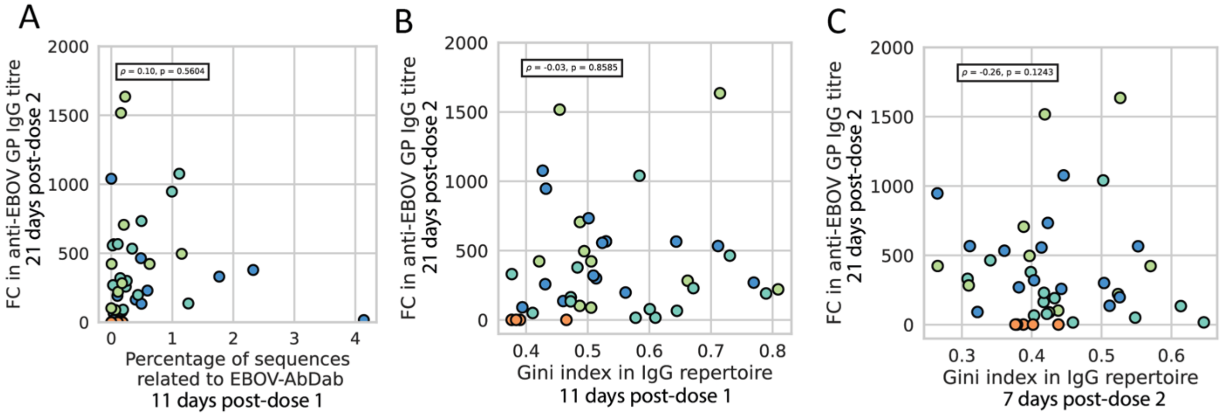

Supplementary Figure 7: there was no significant correlation between the FC in anti-EBOV GP IgG titre with the hit rate to EBOV-AbDab post-dose 1 (**A**), nor with clonal expansion post-dose 1 (**B**) or post-dose 2 (**C**).
